# Integrative Proteogenomics and Single-Cell Transcriptomics Prioritize Candidate Causal Proteins and Therapeutic Targets in Age-Related Macular Degeneration

**DOI:** 10.3390/ijms27188103

**Published:** 2026-09-11

**Authors:** Lei Wen, Fangran Li, Yuan Liu, Ka Zhang, Aiqin Mao, Liangju Liu, Xiaowang Lv, Li Geng, Fan Yu, Lei Feng, Hao Kan

**Affiliations:** 1 Wuxi School of Medicine, Jiangnan University, Wuxi 214122, China; 2 School of Food Science and Technology, Jiangnan University, Wuxi 214122, China

**Keywords:** age-related macular degeneration, protein quantitative trait loci, proteome-wide association studies, summary-data-based mendelian randomization, single-nucleus RNA sequencing

## Abstract

Age-related macular degeneration (AMD) is a major cause of irreversible visual impairment, yet identifying effector proteins and tissue-specific mechanisms underlying genome-wide association study (GWAS) loci remains challenging. This study aimed to systematically prioritize candidate causal circulating proteins and delineate their cellular and transcriptional dynamics in AMD. We integrated plasma protein quantitative trait loci (pQTL) summary statistics from the UK Biobank Pharma Proteomics Project (UKB-PPP; N=53,022) with FinnGen AMD GWAS data using proteome-wide association studies (PWAS), summary-data-based Mendelian randomization (SMR) with the HEIDI test, and Bayesian colocalization analysis. Prioritized candidates were mapped across human and murine retinal single-cell/single-nucleus RNA sequencing atlases. Transcriptional responsiveness was validated in an independent clinical microarray dataset (GSE103060) and in human retinal pigment epithelial cells (ARPE-19) via in vitro inflammatory stimulation and RT-qPCR. Target tractability was assessed using pharmacological databases. Multi-stage genetic screening prioritized five candidate proteins stratified into two confidence tiers: three Tier 1 causal drivers supported by colocalization (PP4 > 0.80)—including risk factors CSF2, IL20RB, and WARS1 (also known as WARS)—alongside two Tier 2 candidates supported by SMR and HEIDI, comprising risk factor PILRA and inversely associated metabolic factor ACADSB. Retinal transcriptomic mapping localized *PILRA* specifically to microglia, *ACADSB* to inner retinal neurons, and *WARS1* to photoreceptors, RPE, and vascular compartments, while *IL20RB* and *CSF2* exhibited low baseline expression. In independent validation cohorts, *IL20RB* and *WARS1* were significantly up-regulated in choroidal neovascularization (CNV) membrane-derived RPE from patients with AMD (p<0.01). Exposure of ARPE-19 cells to TNF-α markedly induced mRNA levels of *IL20RB* (P=0.0025) and *WARS1* (p<0.0001). Dual normalization against ACTB as a secondary internal reference yielded consistent significant induction. Pathway enrichment highlighted cytokine-driven receptor cascades (JAK-STAT signaling) and mitochondrial substrate catabolism (branched-chain amino acid and fatty acid metabolism). Drug–target profiling identified small molecules and nutraceuticals interacting with ACADSB, CSF2, and WARS1. By combining large-scale plasma proteomic genetics with single-cell mapping and experimental validation, this study identifies a prioritized set of candidate causal proteins linking neuroimmune activation, vascular remodeling, and mitochondrial bioenergetics in AMD, providing candidate entry points for mechanistic and therapeutic exploration.

## 1. Introduction

Age-related macular degeneration (AMD) is a progressive neurodegenerative disease of the central retina and a leading cause of irreversible visual impairment in older adults worldwide [1,2]. Clinically, AMD progresses from early asymptomatic stages characterized by sub-retinal pigment epithelium (RPE) extracellular deposits (drusen) to late-stage manifestations: neovascular (wet) AMD, defined by choroidal neovascularization (CNV), or geographic atrophy (GA; advanced dry AMD), characterized by the continuous loss of photoreceptors, RPE, and underlying choriocapillaris [3,4,5]. While anti-vascular endothelial growth factor (anti-VEGF) biologics have substantially improved visual outcomes in neovascular AMD, clinical challenges remain, including variable treatment responsiveness, subretinal fibrosis, and the burden of recurrent intravitreal injections [6]. For GA, recently approved complement inhibitors offer modest reductions in lesion enlargement rates without restoring lost visual acuity, and may carry secondary risks of inflammatory or exudative conversion [7,8]. Consequently, identifying upstream molecular determinants and modifiable pathogenic pathways that govern both inflammatory and metabolic homeostasis in the retinal microenvironment remains an important objective.

AMD has a strong genetic basis, with heritability estimates ranging from 46% to 71% [9]. Large-scale genome-wide association studies (GWAS) have identified dozens of susceptibility loci, underscoring the contributions of complement regulation (e.g., *CFH*, *C3*, *CFI*), lipid transport (e.g., *APOE*, *LIPC*), and extracellular matrix remodeling (e.g., *ARMS2*/*HTRA1*) [10,11,12]. Nonetheless, translating statistical loci into actionable pathophysiological mechanisms faces well-recognized hurdles. The vast majority of AMD-associated variants reside in non-coding regions and are presumed to influence disease susceptibility by altering transcriptional or translational abundance rather than protein-coding sequences [13,14]. Furthermore, dense linkage disequilibrium (LD) blocks frequently span multiple adjacent genes, complicating the identification of true causal effector genes and the determination of the direction of regulatory effect.

Integrative proteogenomic strategies provide a systematic framework to address these limitations [15,16]. Because circulating proteins represent proximate mediators of physiological and pathological processes—and constitute the major class of therapeutic targets—evaluating the genetic regulation of the plasma proteome (protein quantitative trait loci, pQTLs) enables the direct investigation of genotype–protein–phenotype axes [17,18]. Proteome-wide association studies (PWAS) leverage cis-pQTL weights to impute protein abundance into disease GWAS cohorts, prioritizing protein-trait associations while reducing bias from environmental confounding and reverse causation [19,20]. Within this framework, summary-data-based Mendelian randomization (SMR) coupled with the heterogeneity in dependent instruments (HEIDI) test distinguishes potential causal pleiotropy from linkage-mediated confounding [21]. Complementing these methods, Bayesian colocalization analysis formally estimates the probability that GWAS risk variants and pQTLs share a common causal variant within a localized genomic window [22,23].

Previous proteomic association studies in ocular diseases were frequently limited by constrained proteomic depth and sample size. In this study, we leveraged cis-pQTL summary data from the UK Biobank Pharma Proteomics Project [24] (UKB-PPP; N=53,022), profiling nearly 3000 plasma proteins, and integrated these with FinnGen AMD GWAS statistics. We implemented a multi-stage prioritization pipeline combining PWAS, SMR/HEIDI filtering, and high-stringency Bayesian colocalization (${\mathrm{PP}}_{4}>0.80$). To characterize the spatial and cellular context of prioritized candidates, we mapped their expression across human and murine retinal single-cell and single-nucleus transcriptomic atlases. Finally, candidate targets were evaluated in independent human clinical microarray datasets (GSE103060) and validated through in vitro cellular perturbation assays. This integrated workflow prioritizes a focused set of candidate proteins—stratified into Tier 1 causal drivers (CSF2, IL20RB, and WARS1 [historically denoted as WARS]) and Tier 2 candidate mediators (PILRA and ACADSB)—providing targeted insights into neuroimmune modulation and mitochondrial substrate metabolism in AMD.

## 2. Results

**Figure 1:**
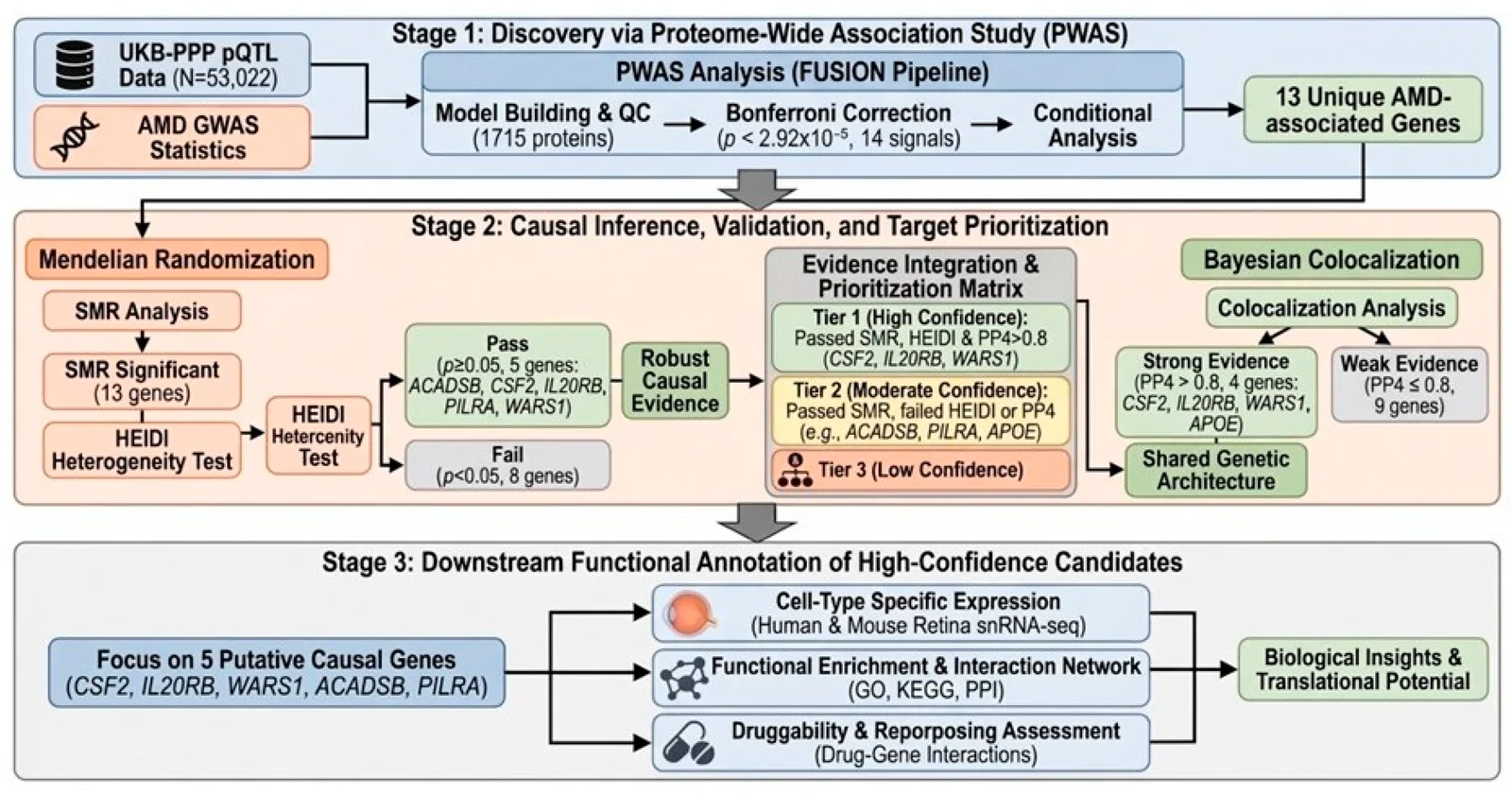
Multi-stage integrative framework for identifying and prioritizing causal proteins for AMD.

The overall analytical workflow is depicted in Figure 1. To prioritize putative causal proteins associated with age-related macular degeneration (AMD), we implemented an integrative framework combining proteome-wide association studies (PWAS), summary-data-based Mendelian randomization (SMR) with the HEIDI heterogeneity test, and Bayesian colocalization analysis.

### 2.1. Identification of AMD-Associated Proteins via PWAS

**Figure 2:**
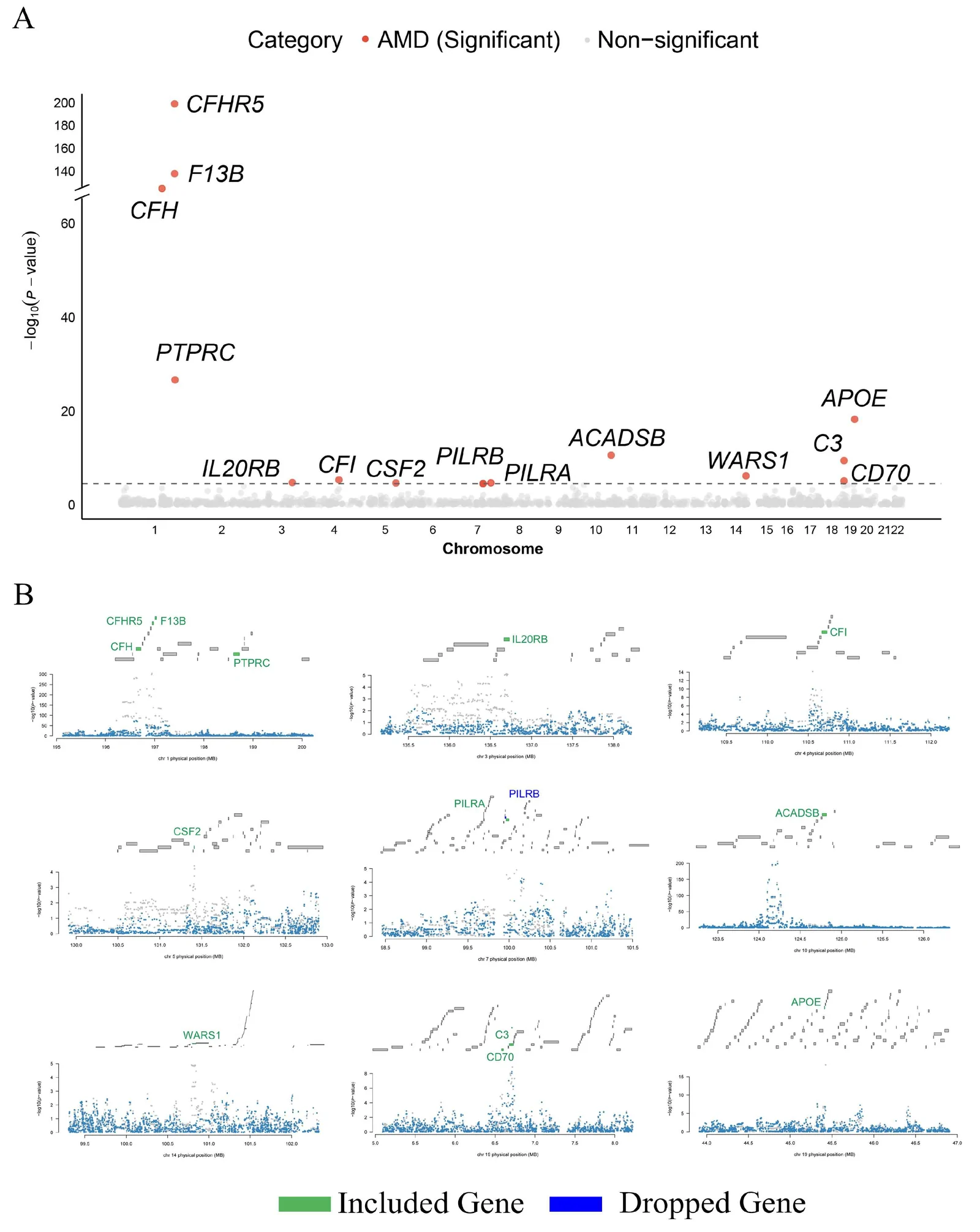
Results of the PWAS analyses. (**A**) Manhattan plot for the PWAS results for AMD. The *x*-axis represents the chromosomal position, and the *y*-axis indicates the significance level (−log_10_*p*-value). Significant genes passing the Bonferroni correction threshold (dashed line) are highlighted in red and labeled with gene symbols. Gray dots represent non-significant associations. Note that the *y*-axis ($-\log_{10}\left(p\right)$) features an axis break for values >60 to appropriately compress extreme genetic association signals while maintaining visual resolution for sub-threshold loci. (**B**) Regional association plots for the identified risk loci following conditional analysis. The plots visualize the prioritization of causal genes within significant genomic regions. Genes highlighted in green (Included Gene) represent the prioritized candidates included in the credible set, while genes in blue (Dropped Gene) were filtered out due to high correlation (linkage disequilibrium) or lack of independent evidence.

Using human plasma pQTL data from the UK Biobank Pharma Proteomics Project (UKB-PPP; N=53,022) [24], cis-genetic prediction models were constructed for 2923 proteins. A total of 1715 proteins exhibited significant SNP-based heritability (P<0.05, $h^{2}>0$; Supplementary Table S1) and were included in downstream PWAS. Integrating these genetically predicted proteomic profiles with AMD genome-wide association study (GWAS) summary statistics (Supplementary Table S2) using the FUSION pipeline [25] identified 14 significant protein-trait associations after Bonferroni correction ($P<2.92\times 10^{-5}$; Figure 2A, Table 1, and Supplementary Table S3). Notably, PILRA reached marginal significance ($P=2.85\times 10^{-5}$) under the optimal Elastic Net model ($R^{2}=0.114$), with consistent effect directions observed across alternative predictive models (LASSO, GBLUP, and BSLMM). Subsequent conditional analysis resolved LD confounding across 7 genes residing within 3 shared multi-signal loci (located within 1 Mb of each other: chr1: CFH/CFHR5/F13B; chr7: PILRA/PILRB; chr19: C3/CD70), filtering out PILRB due to collinearity ($P_{\mathrm{joint}}=0.87$) and retaining 13 conditionally independent candidate proteins significantly associated with AMD (Figure 2B and Supplementary Table S4).

### 2.2. SMR and Colocalization Analyses Prioritize High-Confidence Causal Candidates

**Figure 3:**
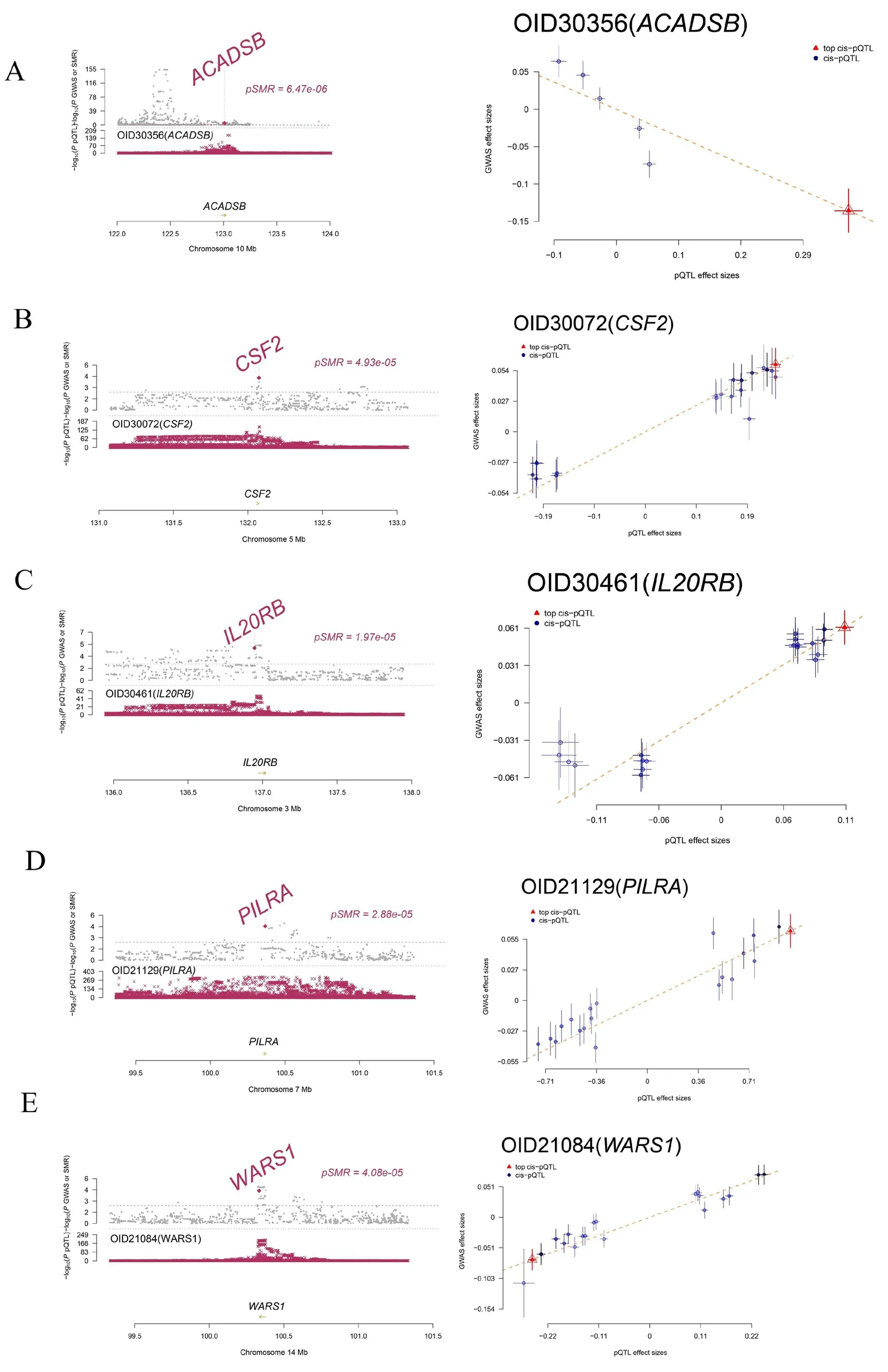
Results for the causal genes. SMR results for five putative causal proteins: (**A**) ACADSB, (**B**) CSF2, (**C**) IL20RB, (**D**) PILRA, and (**E**) WARS1. Left panels: regional association plots showing the colocalization of GWAS signals for AMD (top plot, gray dots) and cis-pQTL signals for the respective plasma protein (bottom plot, maroon dots). The *x*-axis represents the genomic position, and the *y*-axis represents the −log_10_(*p*-value). The top SMR SNP is highlighted with a diamond. Right panels: effect size plots visualizing the relationship between the genetic effect on protein abundance (*x*-axis, cis-pQTL beta) and the genetic effect on AMD risk (*y*-axis, GWAS beta). Each point represents a genetic instrument (SNP). The orange dashed line indicates the estimated causal effect (slope) from the SMR test. Error bars denote standard errors. Blue dots represent cis-pQTLs, and the red triangle highlights the top cis-pQTL instrument. OID represents the Olink Identifier (assay target ID assigned by the Olink Explore 3072 platform in the UKB-PPP study). The dashed gray line represents the multiple-testing threshold (*p* = 0.05/13 = 0.00385), and exact gene-level P_SMR values are reported.

To evaluate whether the associations between the 13 candidate proteins and AMD were driven by potential causality or pleiotropy rather than LD, we performed SMR analysis alongside the HEIDI test [26]. While all 13 proteins showed significant association in the SMR test (Supplementary Table S5), the HEIDI test indicated that associations for 8 proteins were likely confounded by LD ($P_{\mathrm{HEIDI}}<0.05$). Five proteins—ACADSB, CSF2, IL20RB, PILRA, and WARS1 (indexed by their respective Olink Proteomics Identifiers [OIDs] in Figure 3)—passed both thresholds ($P_{\mathrm{SMR}}<0.05$ and $P_{\mathrm{HEIDI}}\geq 0.05$), supporting potential causal associations with AMD (Figure 3A–E and Table 1). Instrumental strength evaluation showed that top cis-pQTL instruments across all 13 candidates exhibited exceptionally high F-statistics (range: 171.4 to 15,464.7; Supplementary Table S5), with the five prioritized candidates ranging from 195.1 (IL20RB) to 15,464.7 (PILRA), confirming that our causal inferences are not confounded by weak-instrument bias. Genetically predicted higher plasma levels of CSF2, IL20RB, PILRA, and WARS1 were positively correlated with AMD risk, whereas ACADSB exhibited an inverse association (Table 1).

**Figure 4:**
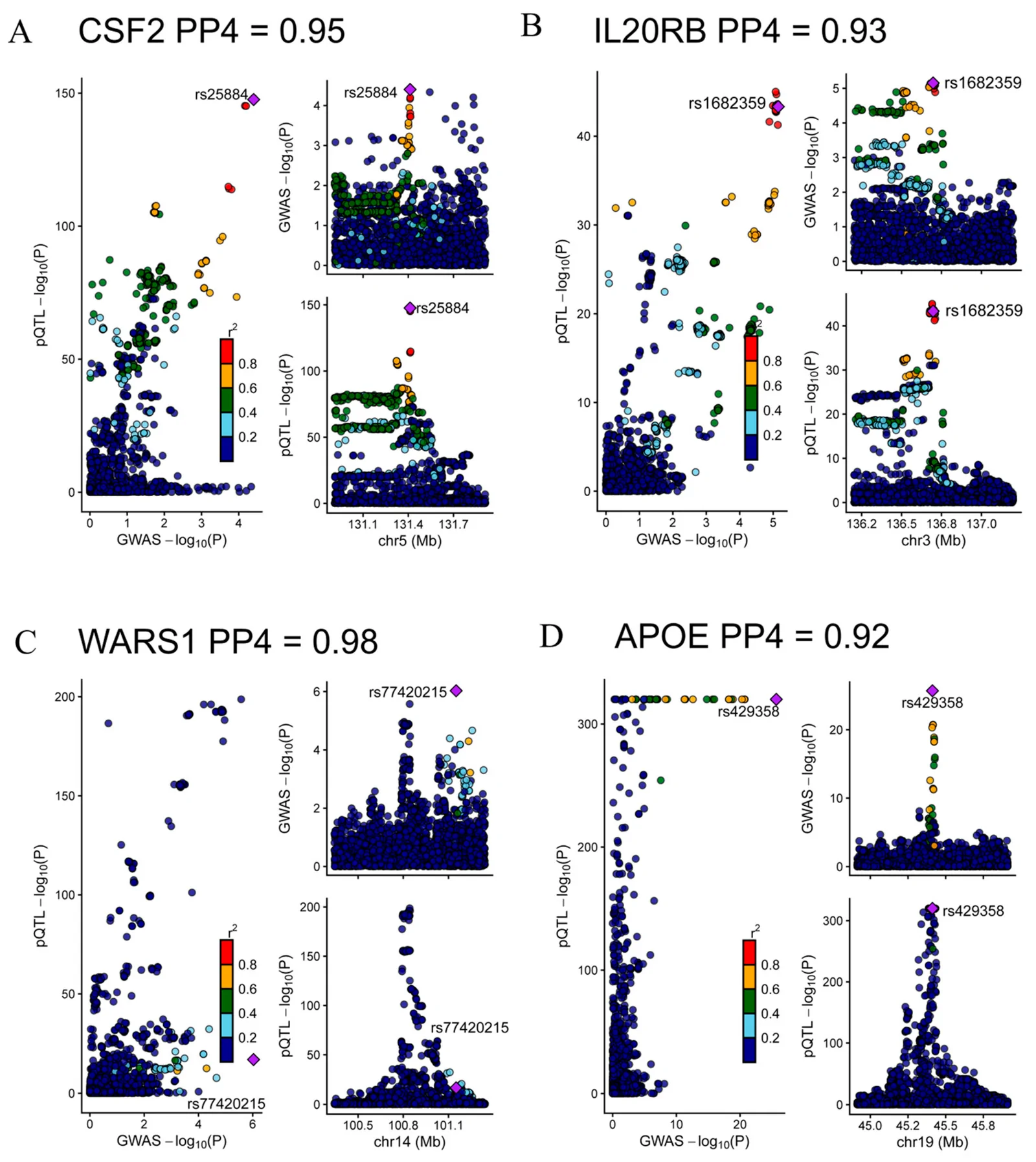
Bayesian colocalization analysis of AMD GWAS signals and plasma pQTLs. LocusCompare plots visualizing the colocalization events for four significant genes (**A**) CSF2, (**B**) IL20RB, (**C**) WARS1 and (**D**) APOE. For each panel, the left plot shows the correlation between the significance of the GWAS association (*y*-axis, −log_10_*P*) and the pQTL association (*x*-axis, −log_10_*P*) for all variants in the region. The right plots display the regional Manhattan plots for GWAS (top) and pQTL (bottom) data. The purple diamond represents the lead SNP for each locus (labeled with rsID). Other SNPs are colored based on their linkage disequilibrium (r^2^) with the lead SNP. The posterior probability for a shared causal variant (PP4) is indicated above each panel, with a threshold of PP4 > 0.8 considered strong evidence of colocalization.

Subsequent Bayesian colocalization analysis assessed whether GWAS risk signals and plasma pQTLs shared a single causal variant within respective loci. Three proteins—CSF2, IL20RB, and WARS1—alongside the established control gene APOE, demonstrated strong colocalization evidence with a posterior probability for hypothesis 4 (${\mathrm{PP}}_{4}$) exceeding 0.80 (Figure 4A–D and Table 1). Integrating these statistical lines of evidence, candidate proteins were prioritized into three distinct tiers: (1) Tier 1 (high confidence: CSF2, IL20RB, and WARS1), which met all SMR, HEIDI, and high-stringency colocalization criteria ($PP_{4}>0.80$), supporting a shared causal variant; (2) Tier 2 (moderate confidence: ACADSB and PILRA), demonstrating robust SMR and HEIDI associations but lacking definitive colocalization support ($PP_{4}\leq 0.80$), warranting consideration as putative mediators subject to future fine-mapping; and (3) Tier 3 (low confidence: CFHR5, F13B, CFH, PTPRC, C3, CFI, and CD70), which failed HEIDI or colocalization filters due to pervasive LD confounding (Table 1).

### 2.3. Cell-Type-Resolved Expression of Prioritized Genes in Human and Mouse Retina

**Figure 5:**
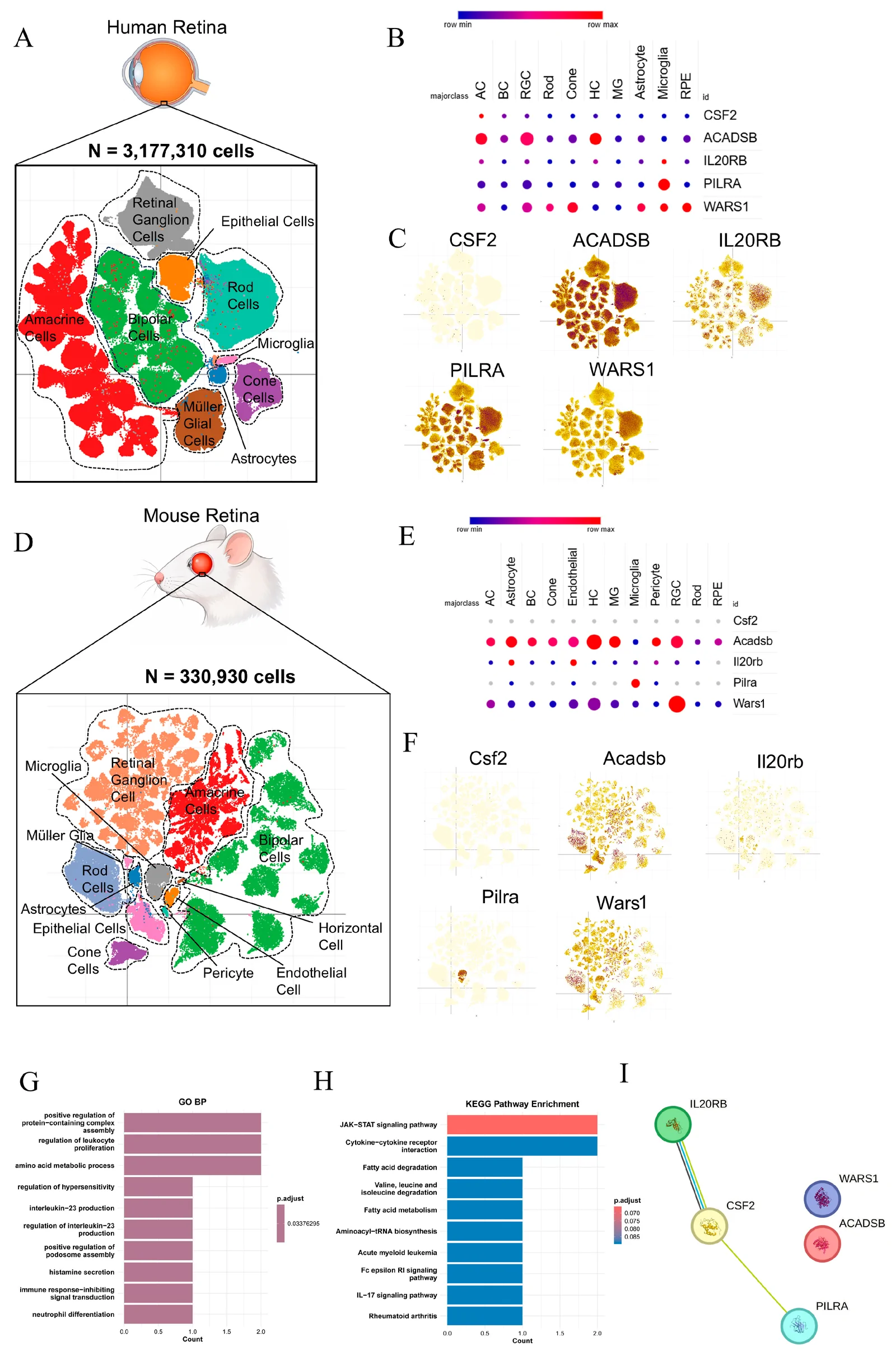
Single-cell transcriptomic profiling and functional characterization of putative AMD causal genes. (**A**–**C**) Expression analysis in the human retina. (**A**) UMAP projection of 3,177,310 cells from the Human Retina Cell Atlas (HRCA), colored by major cell class. (**B**) Dot plot summarizing the expression of the five prioritized genes (*CSF2*, *ACADSB*, *IL20RB*, *PILRA*, *WARS1*) across human retinal cell types. The dot size represents the percentage of cells expressing the gene, and the color intensity (blue to red) indicates the average scaled expression level. (**C**) Feature plots visualizing the spatial expression patterns of the candidate genes on the human UMAP embedding. (**D**–**F**) Cross-species validation in the mouse retina. (**D**) UMAP visualization of 330,930 cells from the Mouse Retina Cell Atlas (MRCA). (**E**) Dot plot and (**F**) feature plots showing the expression of murine orthologs (*Csf2*, *Acadsb*, *Il20rb*, *Pilra*, *Wars1*) in mouse retinal cell lineages. Note the conserved microglia-specific expression of Pilra and the broad neuronal expression of Acadsb. (**G**–**I**) Functional enrichment and interaction networks. (**G**) Gene Ontology (GO) enrichment analysis for Biological Processes (BP). (**H**) KEGG pathway enrichment analysis. Bar lengths represent gene counts, and colors indicate statistical significance (*p*-adjust). (**I**) Protein–Protein Interaction (PPI) network constructed using STRING, revealing a functional cluster involving immune-related proteins (IL20RB, CSF2, PILRA) and distinct metabolic nodes (WARS1, ACADSB). AC, Amacrine cells; BC, Bipolar cells; RGC, Retinal ganglion cells; HC, Horizontal cells; MG, Müller glia; RPE, Retinal pigment epithelium.

To profile the cellular distribution of the five prioritized candidates (*ACADSB*, *CSF2*, *IL20RB*, *PILRA*, and *WARS1*), we examined human retinal single-nucleus RNA sequencing (snRNA-seq) datasets comprising 10 distinct cell clusters (N=3,177,310 nuclei; Figure 5A). Transcripts of the five coding genes showed distinct cellular distributions across retinal subpopulations (Figure 5B,C). *PILRA* was predominantly detected in microglia. *ACADSB* displayed higher expression across neuronal lineages, particularly in amacrine cells, retinal ganglion cells (RGCs), and horizontal cells. *WARS1* exhibited broader expression, with relative enrichment in cone photoreceptors, RGCs, and the retinal pigment epithelium (RPE). In contrast, baseline expression of *CSF2* and *IL20RB* remained low across primary quiescent retinal cell types under physiological conditions (Figure 5B,C).

In the Mouse Retina Cell Atlas (N=330,930 cells; Figure 5D), cellular expression patterns were largely concordant (Figure 5E,F). Pilra showed specific localization to microglia, Acadsb was enriched across inner retinal neurons, and Wars1 was detected in both RGCs and retinal vascular compartments (endothelial cells and pericytes). Transcripts for *Csf2* and *Il20rb* were similarly sparse in resting murine retinal tissue (Figure 5E,F).

### 2.4. Functional Pathway Enrichment and Protein Interaction Networks

Gene Ontology (GO) and KEGG enrichment analyses indicated that the five prioritized genes were enriched in immune-related and metabolic pathways (Figure 5G,H, Supplementary Tables S6 and S7). GO biological process terms included ‘regulation of leukocyte proliferation’, ‘interleukin-23 production’, and ‘immune response-inhibiting signal transduction’, alongside lipid and amino acid metabolic terms. KEGG pathway mapping highlighted the ‘JAK-STAT signaling pathway’, ‘Cytokine-cytokine receptor interaction’, and branched-chain amino acid degradation pathways. Protein–protein interaction (PPI) network modeling identified connected functional nodes between IL20RB, CSF2, and PILRA, whereas WARS1 and ACADSB mapped as distinct nodes consistent with their canonical metabolic and translation-associated roles (Figure 5I).

### 2.5. Transcriptomic and In Vitro Experimental Validation of IL20RB and WARS1

**Figure 6:**
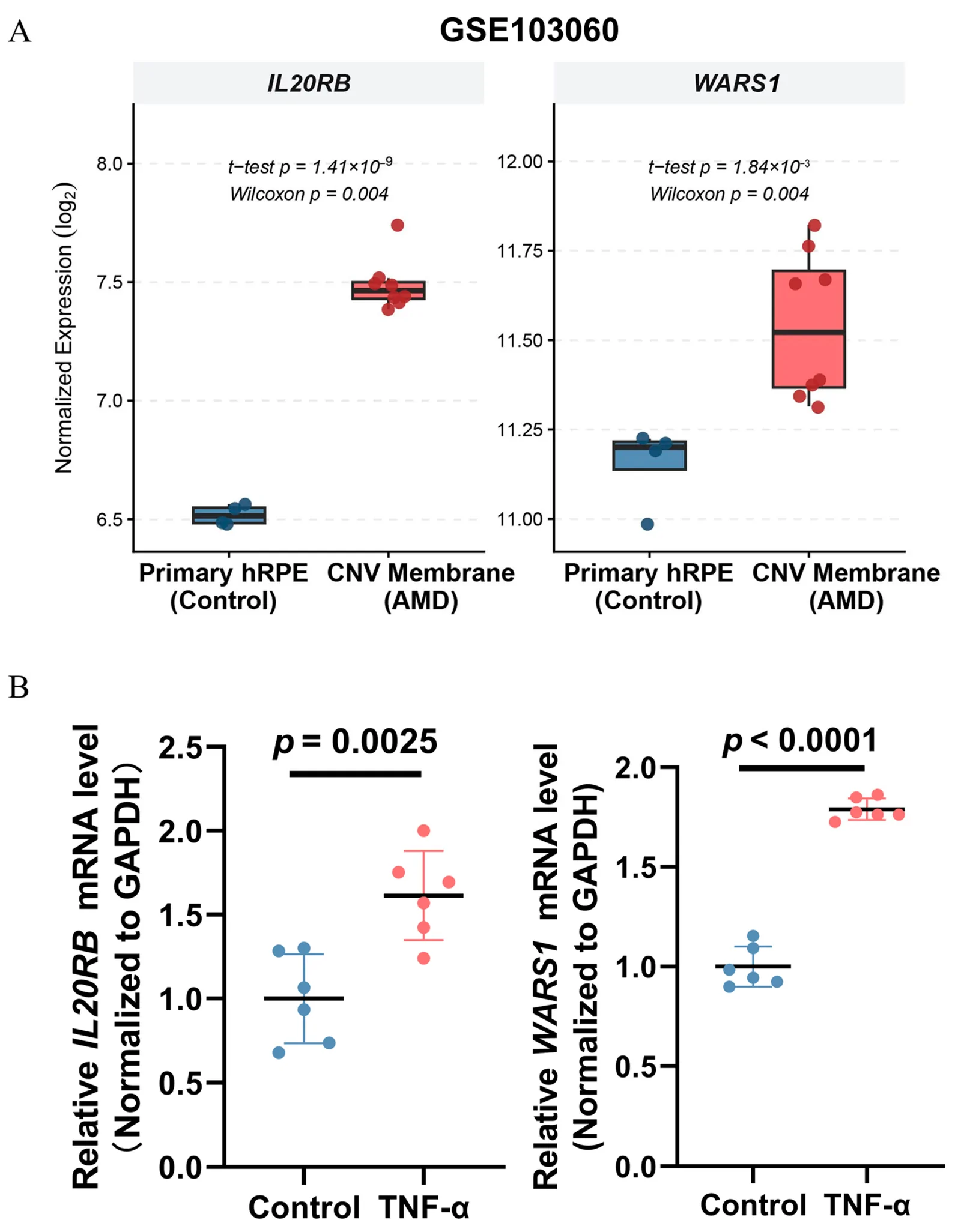
Transcriptomic and in vitro validation of elevated IL20RB and WARS1 expression in AMD. (**A**) Normalized log_2_ expression levels of *IL20RB* and *WARS1* in the GSE103060 dataset comparing primary human retinal pigment epithelium (hRPE, control, *n* = 4) with choroidal neovascularization (CNV) membrane-derived RPE from patients with AMD (*n* = 8). In box plots, horizontal center lines represent the median, box bounds indicate the interquartile range (IQR, 25th-75th percentiles), and whiskers denote 1.5 × IQR; individual data points are overlaid. Statistical significance was evaluated using Welch’s *t*-test and corroborated by exact non-parametric Mann–Whitney U tests (*p* = 0.0040 for both targets) to account for modest sample sizes and non-normal distributions. (**B**) Real-time quantitative PCR (RT-qPCR) analysis of relative *IL20RB* and *WARS1* mRNA expression levels in ARPE-19 cells treated with vehicle control or TNF-α. Data are normalized to internal control and presented as mean ± s.d. (*n* = 6 biological replicates per group). Exact *p* values are indicated above the brackets and calculated using two-tailed unpaired Student’s *t*-test.

To validate the expression changes in prioritized Tier 1 targets in clinical tissue and cellular models, we examined independent human transcriptomic data and performed in vitro assays (Figure 6). In the GSE103060 microarray dataset, normalized $\log_{2}$ expression levels of both *IL20RB* and *WARS1* were significantly elevated in choroidal neovascularization (CNV) membrane-derived RPE cells from patients with neovascular AMD (n=8) compared to primary human RPE controls (n=4; *IL20RB*, parametric *p* = 1.41 × 10^−9^, Mann–Whitney U test *p* = 0.004; *WARS1*, parametric *p* = 1.84 × 10^−3^, Mann–Whitney U test *p* = 0.004; Figure 6A).

To corroborate these findings in a controlled in vitro setting, ARPE-19 cells were challenged with the pro-inflammatory cytokine TNF-α. Real-time quantitative PCR (RT-qPCR) revealed a significant induction of both *IL20RB* (p=0.0025) and *WARS1* (p<0.0001) mRNA levels relative to vehicle-treated control cells (Figure 6B), consistent with their up-regulation under pathological inflammatory conditions observed in AMD tissues. To ensure rigorous quantification and rule out potential reference-gene drift under inflammatory stress, we verified that the raw cycle threshold (Ct) values of the primary reference GAPDH (*p* = 0.54) and the secondary reference ACTB (*p* = 0.49) remained remarkably stable across treatment conditions (Supplementary Figure S1A). Furthermore, secondary normalization against ACTB independently validated the robust induction of both IL20RB (*p* = 0.0004) and WARS1 (*p* < 0.0001) (Supplementary Figure S1B,C), confirming that the observed up-regulation is biologically robust and not an artifact of housekeeping gene variation.

### 2.6. Evaluation of Drug–Gene Interactions

Cross-referencing the prioritized targets against pharmacological databases identified existing small molecules and nutraceuticals interacting with three of the five genes (*ACADSB*, *CSF2*, and *WARS1*; Supplementary Table S8). ACADSB interacts with valproic acid and its metabolic substrate isoleucine. WARS1 is cataloged to interact with its native substrate tryptophan and related chemical derivatives, while CSF2 interacts with talimogene laherparepvec and KB002. These substrate interactions represent mechanism-agnostic biochemical bindings rather than directionally validated therapeutic agents. No direct pharmacological agents were identified for PILRA or IL20RB, pointing to their potential as targets for de novo investigation.

## 3. Discussion

In this study, we integrated large-scale plasma proteomic pQTL datasets with AMD GWAS summary statistics to prioritize candidate causal circulating proteins involved in AMD pathogenesis. Through systematic PWAS, SMR, and Bayesian colocalization screening, candidate proteins were prioritized into Tier 1 causal drivers (CSF2, IL20RB, and WARS1) and Tier 2 putative targets (PILRA and ACADSB, which showed SMR significance without concurrent PP_4_ > 0.8 colocalization).

Complementing classical complement-centric paradigms, our findings, supported by retinal single-cell resolution and independent experimental validations, implicate a coordinated immune-metabolic axis encompassing microglial receptor modulation (PILRA), cytokine signaling (CSF2, IL20RB), non-canonical inflammatory/angiogenic regulation (WARS1), and mitochondrial bioenergetics (ACADSB).

A notable finding from our genetic prioritization is the inverse association between plasma ACADSB levels and AMD risk [27,28]. ACADSB catalyzes a critical dehydrogenation step in mitochondrial fatty acid β-oxidation and branched-chain amino acid (BCAA; specifically isoleucine) catabolism [29]. Given that the retina exhibits exceptionally high metabolic activity and ATP requirements to support phototransduction, age-dependent mitochondrial decay in the retinal pigment epithelium (RPE) and neuroretina is considered a central feature of dry AMD pathology [30]. Single-nucleus transcriptomic mapping localized *ACADSB* primarily to inner retinal neuronal populations, including amacrine cells, retinal ganglion cells (RGCs), and horizontal cells. Reduced ACADSB activity may impair BCAA turnover, promoting the accumulation of toxic intermediate acyl-CoA species and compromising cellular bioenergetics under oxidative stress [31]. Genetically sustained ACADSB expression might support metabolic resilience against age-related energetic deficits by facilitating alternative substrate oxidation. Although drug–target profiling identified valproic acid (VPA) as an ACADSB interactor, retinal responses to VPA remain heterogeneous across differing concentrations and pathophysiological contexts [32]. Dedicated pharmacological modulation of ACADSB or its downstream metabolic nodes will be necessary to evaluate targeted metabolic interventions. In addition, the possibility of reverse causation warrants careful consideration. While the Mendelian randomization framework inherently mitigates confounding and reverse causality by leveraging germline genetic variants fixed at conception, the profound neurodegenerative and metabolic decay accompanying advanced AMD could theoretically perturb circulating acyl-carnitines or mitochondrial enzymes. Nevertheless, the fact that genetically proxied higher ACADSB abundance correlates with reduced AMD risk across individuals without prior disease manifestations strongly argues for a primary protective effect rather than secondary downstream remodeling. Future longitudinal cohorts and bidirectional MR studies will be valuable to formally exclude any latent reverse regulatory feedback across distinct disease stages.

Our results also indicate a positive genetic association between PILRA levels and AMD risk, with transcript expression restricted to microglia in both human and murine retinas [33]. PILRA is an inhibitory receptor bearing immunoreceptor tyrosine-based inhibitory motifs (ITIMs) that attenuates myeloid cell activation upon ligand binding [34]. In neurodegenerative contexts such as Alzheimer’s disease, loss-of-function variants in *PILRA* have been reported to confer protection, likely by relieving baseline suppression on microglial phagocytosis and debris clearance [35]. In the aging retina, sustained clearance of extracellular sub-RPE deposits (drusen) requires homeostatic yet active microglial surveillance. Elevated PILRA expression may elevate the threshold for microglial activation, reducing lipid and protein clearance efficiency and facilitating chronic sterile inflammation. Modulating the PILRA inhibitory axis represents a potential immunotherapeutic strategy to restore microglial surveillance in early or dry AMD. However, given that PILRA was borderline significant in discovery PWAS ($p=2.85\times 10^{-5}$) and lacked Bayesian colocalization support (${\mathrm{PP}}_{4}=0.062$), its classification as a Tier 2 candidate warrants caution, and future functional studies will be necessary to definitively confirm its regulatory contribution.

Beyond its canonical role in ribosomal translation, secreted full-length WARS1 acts as an extracellular signaling factor capable of activating Toll-like receptor (TLR2/TLR4) cascades in macrophages and epithelial cells, stimulating pro-inflammatory cytokine production [36]. While truncated WARS1 fragments (e.g., T2-TrpRS) exhibit anti-angiogenic properties, intact full-length WARS1 has been implicated in inflammatory signaling and vascular remodeling [37,38]. In our study, *WARS1* displayed broad retinal expression, including detection in cone photoreceptors, RPE, and vascular endothelial cells. Supporting these genetic predictions, independent transcriptomic analysis of the GSE103060 microarray dataset demonstrated substantial up-regulation of *WARS1* in human surgically excised choroidal neovascularization (CNV) membranes compared to primary RPE controls (Figure 6A). Furthermore, exposure of human ARPE-19 cells to TNF-α induced a pronounced increase in *WARS1* mRNA expression (Figure 6B), corroborating its sensitivity to the local inflammatory milieu and suggesting that elevated WARS1 may contribute to pathological retinal inflammation and vascular progression.

Similarly, genetic prioritization of CSF2 and IL20RB highlights the contribution of chronic cytokine-driven signaling in AMD progression [39]. IL20RB functions as a shared subunit for the IL-19, IL-20, and IL-24 receptor complexes, engaging the downstream JAK-STAT signaling pathway. Aberrant JAK-STAT activation in retinal tissue has been linked to reactive gliosis, microglial pro-inflammatory polarization, and barrier compromise. In concordant validation, *IL20RB* expression was markedly up-regulated in human CNV-derived RPE tissues (Figure 6A) and was significantly induced in ARPE-19 cells upon pro-inflammatory TNF-α stimulation (Figure 6B). The close protein–protein interaction connectivity observed among IL20RB, CSF2, and PILRA supports the concept of a functional immune network mediating inflammatory responses across the RPE-choroid interface.

Several methodological considerations should be noted. First, although SMR and Bayesian colocalization reduce confounding from horizontal pleiotropy and linkage disequilibrium, these methods rely on circulating plasma pQTLs as proxies. While circulating proteins reflect systemic immune and metabolic states, local protein abundance and isoform-specific regulation within the immune-privileged ocular microenvironment may exhibit tissue-specific differences. Second, the extensive pleiotropy and complex linkage disequilibrium (LD) architecture at specific genomic regions pose inherent challenges for signal deconvolution. In particular, the complement gene cluster on chromosome 1q31.3 (harboring CFH, CFHR5, and F13B) is characterized by dense LD blocks, segmental duplications, and shared regulatory haplotypes. Although we applied GCTA-COJO conditional analysis to isolate multi-signal dependencies across adjacent loci within 1 Mb, statistical conditioning alone has limitations in fully disentangling shared regulatory variants within such a complex locus. Reassuringly, our downstream SMR and HEIDI assessments provided an objective filter, identifying significant heterogeneity (P_HEIDI < 0.05) driven by LD confounding for all candidates in this locus and leading to their conservative categorization as Tier 3 (low confidence). High-resolution long-read sequencing and fine-mapping across diverse cohorts will be essential to definitively resolve distinct causal drivers within this complement hub. Third, our causal inference pipeline utilized the FinnGen R12 cohort to establish a strict two-sample Mendelian randomization design without participant overlap with the UKB-PPP discovery set, thereby avoiding overfitting and weak-instrument bias. However, the Finnish population exhibits unique bottleneck demographics and genetic architecture. Validating these genetic associations in broader, multi-ancestry or pan-European consortia (such as the International AMD Genomics Consortium, IAMDGC) represents an essential next step to ensure generalizability across diverse ethnic backgrounds. Finally, while we validated the transcriptional responsiveness of *IL20RB* and *WARS1* in human clinical tissues and in vitro RPE models (Figure 6), although external validation in GSE103060 confirmed robust expression shifts, the relatively small sample size (n=4 vs. n=8) remains a limitation, warranting further confirmation in larger prospective cohorts. In addition, detailed in vivo loss-of-function and gain-of-function studies will be necessary to establish their exact mechanistic contributions to choroidal neovascularization and photoreceptor degeneration.

In summary, this study integrates plasma proteomic genetics, single-cell transcriptomics, and experimental validation to identify and prioritize candidate causal proteins in AMD. The convergence of findings across Tier 1 drivers (CSF2, IL20RB, WARS1) and Tier 2 candidates (ACADSB, PILRA) offers targeted insights into the metabolic and neuroimmune mechanisms underlying retinal degeneration while clearly distinguishing robustly colocalized targets from candidates requiring further fine-mapping.

## 4. Materials and Methods

### 4.1. Data Sources and Quality Control

Plasma protein quantitative trait loci (pQTL) data: Genetic instruments for circulating plasma protein levels were obtained from the UK Biobank Pharma Proteomics Project (UKB-PPP) European cohort (N=53,022) [24]. Protein relative abundance was measured using the Proximity Extension Assay (PEA)-based Olink Explore 3072 platform across 2923 proteins. Summary statistics for cis-pQTLs within ±500 kb of the transcription start site (TSS) of the respective protein-coding genes were retrieved.

AMD genome-wide association study (GWAS) data: Genome-wide summary statistics for AMD susceptibility were derived from the FinnGen consortium (Release R12; https://www.finngen.fi/en, accessed on 6 August 2026). Standard quality control filters excluded variants with minor allele frequency (MAF) <0.01 or imputation info score <0.30.

### 4.2. Proteome-Wide Association Study (PWAS)

PWAS was performed using the FUSION pipeline [40]. SNP-based cis-heritability ($h^{2}$) was estimated using restricted maximum likelihood (REML). Proteins with significant heritability (p<0.05 and $h^{2}>0$) were retained to train functional weight prediction models (Elastic Net v1.5.2, LASSO v5, and Bayesian Sparse Linear Mixed Model [BSLMM]) [41]. The optimal model with the highest cross-validation $R^{2}$ was utilized to predict protein levels in the AMD GWAS summary cohort. A Bonferroni-corrected significance threshold was defined as $p<0.05/N_{\mathrm{tested}\_\mathrm{proteins}}$. Multi-signal genomic loci (defined as candidate genes with transcription start sites located within a 1 Mb physical window) were evaluated using conditional and joint analysis (GCTA-COJO) to filter out redundant signals caused by LD [42].

### 4.3. Causal Inference Using SMR and the HEIDI Test

To evaluate causal or pleiotropic associations between candidate proteins and AMD, SMR analysis was conducted using the SMR software package (v1.03) [43]. To differentiate whether the observed association arose from a shared causal variant (pleiotropy) or distinct variants in linkage (LD), the HEIDI test was executed using multi-SNP instruments from cis-regions ($P_{\mathrm{HEIDI}}\geq 0.05$ indicating consistent effect sizes across instruments and absence of significant heterogeneity). Putative causal proteins were defined by $P_{\mathrm{SMR}}<0.05/N_{\mathrm{candidates}}$ and $P_{\mathrm{HEIDI}}\geq 0.05$. To assess statistical instrument strength and rule out weak-instrument bias, the F-statistic was calculated for the top cis-pQTL instrument of each protein using $F={\left(b_{\mathrm{pQTL}}/se_{\mathrm{pQTL}}\right)}^{2}$, with phenotypic variance explained estimated as $R^{2}=F/\left(F+N-2\right)$ (where N=53,022). An F-statistic threshold >10 was considered indicative of strong genetic instruments (Supplementary Table S5).

### 4.4. Bayesian Colocalization Analysis

Bayesian colocalization was implemented using the coloc package in R (v5.2.3) across a 1 Mb genomic window surrounding each candidate gene. The analysis evaluated five mutually exclusive hypotheses (*H*_0_ to *H*_4_). Strong evidence of a shared genetic causal variant between plasma protein abundance and AMD risk was defined as a posterior probability for hypothesis 4 (${\mathrm{PP}}_{4}$) >0.80, assuming default prior probabilities ($p_{1}=10^{-4}$, $p_{2}=10^{-4}$, $p_{12}=10^{-5}$).

### 4.5. Single-Cell and Single-Nucleus Transcriptomic Mapping

Cell-type-specific retinal expression was assessed using pre-processed and curated single-cell datasets hosted on the Single Cell Portal (Broad Institute, Cambridge, MA, USA). We examined: (1) the Human Retina Cell Atlas (HRCA; snRNA-seq, N=3,177,310 nuclei from 122 human donor eyes) and (2) the Mouse Retina Cell Atlas (MRCA; scRNA-seq, N=330,930 cells from C57BL/6J mice). Normalized expression levels and percentage of expressing cells across pre-annotated major retinal cell populations were extracted directly from the standardized reference atlases.

### 4.6. Pathway Enrichment and Network Analyses

Gene Ontology (GO) biological processes and Kyoto Encyclopedia of Genes and Genomes (KEGG) pathway enrichment analyses were performed using the clusterProfiler package (v4.14.6) in R [44]. Multiple testing correction was applied using the Benjamini–Hochberg method (adjusted P<0.05). Protein–protein interaction (PPI) networks were retrieved from the STRING database (v12.0) with a minimum interaction confidence score threshold set to >0.70.

### 4.7. Microarray Transcriptomic Validation (GSE103060)

Gene expression data from human surgically extracted choroidal neovascularization (CNV) membranes and control retinal pigment epithelium were obtained from the GEO database (accession: GSE103060). The dataset was profiled on the Agilent Whole Human Genome Microarray 4 × 44 K platform (GPL6480). Expression matrices and metadata were imported using GEOquery (v2.74.0). Microarray signal intensities were $\log_{2}$-transformed where appropriate. Primary human RPE cultures (n=4) served as healthy controls, and late-stage CNV membrane-derived RPE cell lines (n=8) represented the AMD phenotype. Differential gene expression was modeled using empirical Bayes moderated t-statistics implemented in the limma package (v3.62.2).

### 4.8. Cell Culture, In Vitro Inflammatory Stimulation, and RT-qPCR Validation

Cell culture and treatment: Human retinal pigment epithelial cells (ARPE-19 [ATCC, Manassas, VA, USA]) were maintained in DMEM medium (Gibco, New York, NY, USA) supplemented with 10% fetal bovine serum (FBS) and 1% penicillin-streptomycin at 37 °C in a humidified incubator containing $5\%\;{\mathrm{CO}}_{2}$. Prior to stimulation, cells were seeded in 6-well plates and synchronized by serum starvation (0.5% FBS) for 16 h. Cells were subsequently exposed to recombinant human TNF-α (20 ng/mL; Yeasen, Shanghai, China) or vehicle control (sterile PBS) for 24 h (n=6 biological replicates per condition).

RNA extraction and RT-qPCR: Total RNA was extracted using TRIzol reagent (CWBIO, Taizhou, China) following the manufacturer’s protocol. RNA concentration and purity were determined by spectrophotometry (NanoDrop 2000, Wilmington, DE, USA). Complementary DNA (cDNA) was synthesized from 1 μg of total RNA using the PrimeScript RT Master Mix (Vazyme, Nanjing, China). Real-time quantitative PCR was performed on a QuantStudio Real-Time PCR System using TB Green Premix Ex Taq II (Vazyme, Nanjing, China). The transcriptional stability of GAPDH under 24 h TNF-α stimulation was verified by evaluating raw Ct values. In addition, ACTB was evaluated as a secondary reference gene, confirming identical induction patterns. Relative mRNA expression was calculated using the $2^{-\Delta \Delta C_{t}}$ method normalized to GAPDH. The specific primer sequences utilized in this study are detailed in Supplementary Table S9.

### 4.9. Druggability and Translational Evaluation

Target tractability and drug–gene interactions were evaluated using the Drug-Gene Interaction Database (DGIdb v5.0) and the Open Targets Platform (https://platform.opentargets.org/, accessed on 10 June 2026) [45]. Identified small molecules, biologics, and clinical compounds were categorized by approval status and mechanism of action, followed by evaluation for concordance with the genetic effect direction (risk vs. protective).

### 4.10. Statistical Analysis

All computational and statistical analyses were performed in R (v4.4.1) and Linux environments. Data normality was assessed using the Shapiro–Wilk test. For the clinical microarray validation (GSE103060) where sample sizes were small (*n* = 4 vs. *n* = 8), two-group comparisons were evaluated using Welch’s *t*-tests alongside exact non-parametric Mann–Whitney U tests (Wilcoxon rank-sum tests) as sensitivity analyses. RT-qPCR quantitative data met normality assumptions and were assessed using two-tailed unpaired Student’s *t*-tests. Data are presented as mean ± standard deviation (s.d.) unless otherwise specified. A p value <0.05 was considered statistically significant.

## 5. Conclusions

In summary, this study systematically integrates large-scale plasma proteomic pQTL data with AMD genome-wide association statistics to prioritize putative causal circulating proteins in AMD pathogenesis. Through multi-tiered genetic filtering (*P*WAS, SMR/HEIDI, and Bayesian colocalization), single-cell transcriptomic mapping, and independent experimental validations, we prioritized Tier 1 causal candidates (CSF2, IL20RB, and WARS1) supported by shared genetic etiology, alongside Tier 2 candidates (PILRA and ACADSB) as plausible biological mediators, differentiating targets with robust colocalization from those warranting cautious interpretation. These findings expand the molecular landscape of AMD beyond canonical complement pathways, highlighting the coordinated involvement of inner retinal mitochondrial substrate catabolism, microglial inhibitory receptor regulation, and cytokine-driven inflammatory signaling. Collectively, our work provides prioritized molecular targets and a functional framework to guide future mechanistic studies and translational investigations in retinal degenerative diseases.

## Figures and Tables

**Table 1:** Summary Results From PWAS, Colocalization, and SMR for 13 PWAS-Identified Proteins.

Protein	Protein Full Name	PWAS		Conditional		Colocalization	SMR			Category
		PWAS.Z	PWAS.*P*	JOINT.Z	JOINT.*P*	PP4 > 0.8	Beta_SMR	*P*_SMR	*P*_HEIDI_SMR	
CFHR5	Complement factor H-related protein 5	30.158	8.42 × 10^−200^	17.6	1.30 × 10^−69^	NO	1.16695	9.966738 × 10^−141^	3.342102 × 10^−162^	Tier3
F13B	Coagulation factor XIII B chain	25.054	1.58 × 10^−138^	−5.4	6.40 × 10^−8^	NO	0.758027	1.831611 × 10^−115^	2.424725 × 10^−127^	Tier3
CFH	Complement factor H	−19.899	4.15 × 10^−88^	−37	3.40 × 10^−306^	NO	−1.03555	3.750797 × 10^−73^	4.499367 × 10^−69^	Tier3
PTPRC	Receptor-type tyrosine-protein phosphatase C	10.8375	2.29 × 10^−27^	10.8	2.30 × 10^−27^	NO	0.403613	2.230248 × 10^−16^	1.363035 × 10^−8^	Tier3
APOE	Apolipoprotein E	8.897	5.74 × 10^−19^	8.9	5.70 × 10^−19^	YES	0.179501	9.089566 × 10^−19^	5.915914 × 10^−3^	Tier2
ACADSB	Short/branched chain specific acyl-CoA dehydrogenase	−6.67107	2.54 × 10^−11^	−6.7	2.50 × 10^−11^	NO	−0.371053	6.470567 × 10^−6^	8.426859 × 10^−2^	Tier2
C3	Complement C3c alpha’ chain fragment 1	6.27128	3.58 × 10^−10^	6.3	3.60 × 10^−10^	NO	0.693906	1.549948 × 10^−7^	3.298321 × 10^−3^	Tier3
WARS1	tryptophanyl-tRNA synthetase 1	4.9756	6.51 × 10^−7^	5	6.50 × 10^−7^	YES	0.276368	4.079768 × 10^−5^	2.958209 × 10^−1^	Tier1
CFI	Complement factor I heavy chain	−4.5864	4.51 × 10^−6^	−4.6	4.50 × 10^−6^	NO	−0.403909	4.532561 × 10^−10^	1.500960 × 10^−4^	Tier3
CD70	CD70 antigen	4.474	7.68 × 10^−6^	4.5	7.70 × 10^−6^	NO	0.0867096	7.837316 × 10^−6^	2.139192 × 10^−3^	Tier3
IL20RB	Interleukin-20 receptor subunit beta	4.30238	1.69 × 10^−5^	4.3	1.70 × 10^−5^	YES	0.547856	1.970210 × 10^−5^	3.466991 × 10^−1^	Tier1
CSF2	Granulocyte-macrophage colony-stimulating factor	4.2595	2.05 × 10^−5^	4.3	2.00 × 10^−5^	YES	0.244493	4.934043 × 10^−5^	4.941016 × 10^−1^	Tier1
PILRA	Paired immunoglobulin-like type 2 receptor alpha	4.185	2.85 × 10^−5^	4.2	2.90 × 10^−5^	NO	0.0622202	2.883881 × 10^−5^	2.195555 × 10^−1^	Tier2
PILRB	Paired immunoglobulin-like type 2 receptor beta	4.185	2.85 × 10^−5^	0.16	0.87	-	-	-	-	-

PP4 > 0.8 means 2 signals were considered to have a strong support of colocalization. PWAS.Z: Z-score from discovery PWAS analysis; PWAS.*P*: *p*-value from discovery PWAS analysis; JOINT.Z: Z-score from conditional analysis; JOINT.*P*: *p*-value from conditional analysis; Beta_SMR: Effect estimate from the top snp SMR analysis; *P*_SMR: *p*-value from the top snp SMR analysis; *P*_HEIDI_SMR: *p*-value from HEIDI test.

## Data Availability

Publicly available datasets were analyzed in this study. This data can be found here: The GWAS summary statistics for AMD from the FinnGen Consortium (https://www.finngen.fi/en, accessed on 6 August 2026); The pQTL data from the UK Biobank Pharma Proteomics Project (https://registry.opendata.aws/ukbppp/, accessed on 6 August 2026); The single-cell RNA sequencing data of ocular tissues (project number: SCP2805 and SCP2560) can be accessed via the Broad Institute Single Cell Portal (https://singlecell.broadinstitute.org/single_cell, accessed on 6 August 2026).

## Supplementary Materials

The following supporting information can be downloaded at: https://www.mdpi.com/article/10.3390/ijms27188103/s1.

## Abbreviations

The following abbreviations are used in this manuscript:

ACADSB	Acyl-CoA dehydrogenase short/branched chain
AMD	Age-related macular degeneration
Anti-VEGF	Anti-vascular endothelial growth factor
BCAA	Branched-chain amino acid
CNV	Choroidal neovascularization
CSF2	Colony-stimulating factor 2
GA	Geographic atrophy
GWAS	Genome-wide association study
HEIDI	Heterogeneity in dependent instruments
HRCA	Human Retina Cell Atlas
IL20RB	Interleukin 20 receptor subunit beta
LD	Linkage disequilibrium
MRCA	Mouse Retina Cell Atlas
OID	Olink Identifier
PILRA	Paired immunoglobulin-like type 2 receptor alpha
PP4	Posterior probability of Hypothesis 4
PPI	Protein–protein interaction
pQTL	Protein quantitative trait locus
PWAS	Proteome-wide association study
RGC	Retinal ganglion cell
RPE	Retinal pigment epithelium
RT-qPCR	Real-time quantitative polymerase chain reaction
SMR	Summary-data-based Mendelian randomization
SNP	Single nucleotide polymorphism
snRNA-seq	Single-nucleus RNA sequencing
TLR	Toll-like receptor
TNF-α	Tumor necrosis factor alpha
UKB-PPP	UK Biobank Pharma Proteomics Project
WARS1 (WARS)	Tryptophanyl-tRNA synthetase 1
