# Supplementary material for: Integrative Proteogenomics and Single-Cell Transcriptomics Prioritize Candidate Causal Proteins and Therapeutic Targets in Age-Related Macular Degeneration
Source: Int J Mol Sci. 2026 Sep 11;27(18):8103. doi: 10.3390/ijms27188103 (PMC13607255; doi:10.3390/ijms27188103)
Supplement: Supplementary file 1 [file ijms-27-08103-s001.zip › Supplementary Materials/supplementary figure.pdf]

Supplementary figure

Integrative proteogenomics and single-cell transcriptomics prioritize candidate causal proteins and therapeutic targets in age-related macular degeneration

Lei Wen[1#], Fangran Li[1#], Yuan Liu[1], Ka Zhang[1], Aiqin Mao[1], Liangju Liu[1], Xiaowang Lv[1], Li Geng[1], Fan Yu[1], Lei Feng[1], Hao Kan[1,2*]

[1] Wuxi School of Medicine, Jiangnan University, Wuxi, Jiangsu, China.
[2] School of Food Science and Technology, Jiangnan University, Wuxi, Jiangsu, China.
* Correspondence, Hao Kan, email: kanhao@jiangnan.edu.cn.
# Lei Wen and Fangran Li contributed equally to this work.

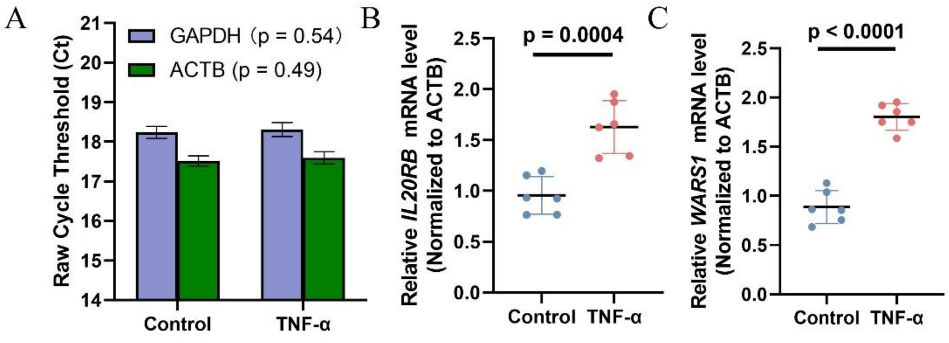

Supplementary Figure S1. Validation of housekeeping gene stability and target expression using secondary reference gene ACTB. (A) Raw cycle threshold (Ct) values of GAPDH and ACTB in ARPE-19 cells treated with vehicle control or TNF-*α* (20 ng/mL, 24 h; n = 6 independent biological replicates). Differences between groups were evaluated by two-tailed unpaired Student's t-test (GAPDH, p = 0.54; ACTB, p = 0.49), confirming exceptional transcriptional stability under inflammatory stimulation. (B–C) Relative mRNA expression levels of *IL20RB* (B) and *WARS1* (C) in ARPE-19 cells normalized against ACTB. Data are presented as mean ± SD, with individual biological replicates shown as distinct dots. Statistical significance was assessed using two-tailed unpaired Student's t-test (*IL20RB*, p = 0.0004; *WARS1*, p < 0.0001).
